# Desmoplastic Small Round Cell Tumors of the Gastrointestinal Tract

**DOI:** 10.3390/cancers16234101

**Published:** 2024-12-07

**Authors:** Jeffrey Gonzalez, Stephanie Ocejo, Mercy Iribarren, Alvaro Abreu, Hisham F. Bahmad, Robert Poppiti

**Affiliations:** 1 Herbert Wertheim College of Medicine, Florida International University, Miami, FL 33199, USA; jgonz1074@med.fiu.edu (J.G.); socej001@med.fiu.edu (S.O.); mirib006@med.fiu.edu (M.I.); aabre070@med.fiu.edu (A.A.); 2 The Arkadi M. Rywlin M.D. Department of Pathology and Laboratory Medicine, Mount Sinai Medical Center, Miami Beach, FL 33140, USA; robert.poppiti@msmc.com; 3 Department of Pathology, Herbert Wertheim College of Medicine, Florida International University, Miami, FL 33199, USA

**Keywords:** DSRCT, sarcoma, *EWSR1::WT1* gene fusion, gastrointestinal tract, Wilms tumor, Ewing sarcoma, rhabdomyosarcoma

## Abstract

Desmoplastic small round cell tumors (DSRCTs) of the gastrointestinal (GI) tract are a rare and highly aggressive variant of soft tissue sarcomas, predominantly affecting the abdominal region. These tumors are believed to originate from multipotent mesenchymal stem cells or primitive progenitor cells. They are composed of small round tumor cells associated with prominent stromal desmoplasia, polyphenotypic differentiation, and *EWSR1::WT1* gene fusion. Diagnostically, DSRCTs present a significant challenge due to their histological resemblance to other small round cell tumors, such as Ewing sarcoma and rhabdomyosarcoma, necessitating the use of ancillary tests, including immunopanels and molecular analysis, to reach a definitive diagnosis. Immunohistochemical staining, including markers like cytokeratin, vimentin, desmin, and WT1, has proven valuable in differentiating DSRCTs from their mimickers. The prognosis of these tumors is highly dependent on factors such as tumor location and stage at diagnosis, and given their aggressive nature, a multidisciplinary approach may be required that combines surgical resection, chemotherapy, and radiation therapy, among other options. In this review, we provide a synopsis of the pathophysiology of DSRCTs and the latest diagnostic advancements, including the utility of molecular profiling and novel biomarkers.

## Simple Summary

Desmoplastic small round cell tumors (DSRCTs) are rare, aggressive malignant neoplasms that typically affect the abdominal region. The challenge in diagnosing those tumors is rendered to their histopathologic resemblance to other small round cell tumors, such as Ewing sarcoma and rhabdomyosarcoma. Our review aims to provide a better understanding of the key histomorphologic and genetic features that make DSRCTs unique, including specific gene fusions. We highlight the importance of ancillary tests, including immunohistochemical staining and molecular profiling, in making an accurate diagnosis. By improving the way these tumors are identified and treated, we hope this review will help guide both pathologists and clinicians and improve outcomes for patients with DSRCTs.

## 1. Introduction

Desmoplastic small round cell tumors (DSRCTs) are a rare and aggressive variant of soft tissue sarcomas of polyphenotypic differentiation [1]. They were first described in 1989 by Gerald and Rosai [2,3]. The incidence of these malignancies is exceedingly low, with an age-adjusted incidence rate in adults of approximately 0.3 per million [4]. In an article published in 2012, it was mentioned that fewer than 200 cases have been reported in the English literature, highlighting the rarity of this tumor [5].

Soft tissue sarcomas, the broader category encompassing DSRCTs, are typically aggressive tumors that often exhibit limited responsiveness to systemic therapies. This challenge is further compounded in small round cell sarcomas, which are generally associated with a poor prognosis due to the ineffectiveness of conventional systemic treatments [6]. For instance, the 5-year survival rate for Ewing sarcoma ranges between 80% and 85% for children younger than 15 years and between 20% and 69% for adolescents aged 15 to 19 years [7,8]. The poor response to therapy is largely attributed to genetic abnormalities within the sarcoma cells, including *EWSR1*::non-*ETS* fusions, *BCOR* genet alterations, and *EWSR1::ETS* fusions. Additionally, these tumors may harbor rare mutations, such as those involving *TP53* and *CDKN2A*, further complicating treatment outcomes [9,10].

DSRCTs are strongly associated with the gastrointestinal (GI) tract, most commonly arising in the abdominal region, including the retroperitoneum, pelvis, omentum, and mesentery [1]. Although predominantly occurring in the abdomen, DSRCTs can occasionally arise in other locations, such as the thoracic cavity and paratesticular region [11,12]. In DSRCTs affecting the GI tract, patients usually present with abdominal pain, abdominal distension, and/or constipation [8,13].

## 2. Pathogenesis and Molecular Characteristics of DSRCTs of the GI Tract

The exact origin of DSRCTs in the GI system is unknown, but they are thought to arise from primitive, multipotent cells that can differentiate into various cell lineages. This is reflected by the broad immunohistochemical profile where DSRCT cells express different epithelial, mesenchymal, and neural markers. These tumors are usually associated with mesothelial surfaces, particularly in the peritoneal cavity that may arise from or have a close relationship to a subset of mesothelial cells. They are aggressive in nature and frequently present with peritoneal dissemination involving abdominal organs [14].

**Figure 1:**
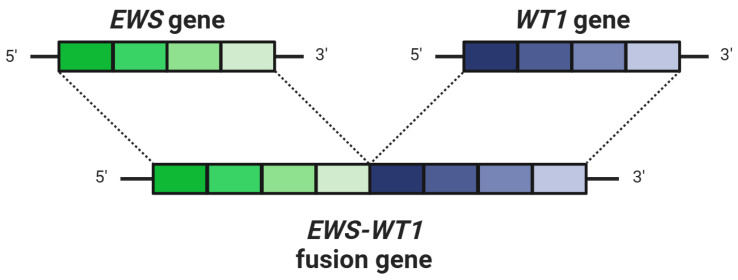
Schematic illustrating the reciprocal translocation t(11;22)(p13;q12), which modulates transcriptional activity and downstream signaling pathways associated with the development of DSRCTs. *WT1* refers to the Wilms tumor gene, *EWS* denotes the Ewing sarcoma gene, and *EWS-WT1* represents the resulting fusion gene.

The molecular basis of DSRCTs involving the GI tract primarily focuses on the *EWSR1::WT1* gene fusion (Figure 1). This key genetic change drives tumor development. It occurs due to a chromosomal rearrangement, specifically t(11;22)(p13;q12), which fuses the 5′ region of the Ewing sarcoma gene (*EWSR1*) with the 3′ DNA-binding domain of the Wilms tumor gene (*WT1*) [15]. This fusion creates a chimeric transcript that produces the *EWS-WT1* fusion protein [16]. The *EWS-WT1* fusion protein functions as a potent transcription factor, significantly impacting several oncogenic pathways that contribute to the development of DSRCTs. It enhances transcriptional activation, leading to the upregulation of target genes that promote cell proliferation, survival, and migration [15,16]. Some of the key upregulated genes include those involved in the cell cycle, such as *CDK4/6*, which promotes cell proliferation, and genes involved in cell adhesion and migration pathways, such as components of the *WNT* signaling pathway. This fusion protein also influences genes involved in Hedgehog and Notch signaling pathways, contributing to oncogenic transformations by altering cellular proliferation and apoptosis control mechanisms [15,17]. This results in uncontrolled tumor growth and resistance to apoptosis, the process of programmed cell death [16]. The *EWS-WT1* fusion protein also interacts with various co-factors and transcriptional regulators, amplifying its cancerous effects and facilitating tumor progression [15,16]. Furthermore, changes in gene expression driven by the *EWS-WT1* fusion protein may help tumor cells evade immune surveillance, allowing them to persist and grow [16].

DSRCTs of the GI tract are believed to arise from multipotent mesenchymal stem or primitive progenitor cells which can become any type of tissue. They exhibit mixed histology, characterized by small round cells in a dense fibrous tissue environment known as desmoplastic stroma [18]. The stroma offers both structural support and shields tumor cells from being recognized and attacked by the immune system [19]. In addition to the common *EWSR1::WT1* fusion, DSRCTs commonly display alterations in other pathways that make the tumor more aggressive and allow it to spread early in its development, prompting tumor metastasis [20].

## 3. Differential Diagnosis of DSRCT of the GI Tract

DSRCTs are among several different types of small round cell tumors affecting the GI tract such as Wilms tumor, Ewing sarcoma, rhabdomyosarcoma, neuroblastoma, and small-cell carcinoma, among others. Although these tumors may appear similar morphologically, their genetic makeup and, thereby, modes of treatment are distinct.

### 3.1. Ewing Sarcoma

Ewing Sarcoma is an aggressive bone tumor primarily affecting children and young adults, frequently associated with *EWSR1* gene fusions. Similar to a DSRCT, it consists of small round cells that can present in the abdominal area. In contrast, Ewing sarcoma usually displays its genetic specificity through the rearrangement of the *EWSR1* gene that results in fusion with *FLI1* to form an oncogenic chimeric protein called EWS-FLI1 [21]. The tumor is commonly CD99 positive, unlike a DSRCT.

### 3.2. Alveolar Rhabdomyosarcoma

Rhabdomyosarcoma is a malignant soft tissue cancer originating from skeletal muscle cells primarily affecting children, with multiple subtypes. Alveolar rhabdomyosarcoma contains a small round cell morphology and may invade the GI tract like DSRCTs [22]. Diagnosis is typically made using muscle differentiation IHC markers myogenin and MyoD1 (myogenic differentiation 1).

### 3.3. Neuroblastoma

Neuroblastoma is a malignant tumor originating from neural crest cells in the abdomen, primarily in infants and young children [23]. It is associated with a characteristic rosette formation by histomorphology and expresses neuroendocrine markers such as synaptophysin and chromogranin [24]. Its prognosis varies depending on the age of onset and tumor stage [23].

### 3.4. Small Cell Carcinoma

Small cell carcinoma is a fast-growing cancer that is mostly found in the lungs of smokers. However, it can also arise within the GI tract. It is composed of small, poorly differentiated cells. It is a high-grade, aggressive tumor. Unlike a DSRCT, it requires a considerable amount of chemotherapy and has a worse overall prognosis [25].

## 4. Histopathological Features of DSRCT of the GI Tract

A DSRCT is characteristically composed of nests of small, round-to-oval, undifferentiated cells with scant eosinophilic cytoplasm and small, hyperchromatic nuclei with inconspicuous nucleoli. These cells are typically arranged in sheets and surrounded by abundant dense desmoplastic fibrous stroma, composed of fibroblasts/myofibroblasts within a loose extracellular matrix or collagen. Indeed, the stroma is collagen-rich and may show myxoid change or hyalinization. Furthermore, DSRCTs rapidly divide, displaying high mitotic activity and large areas of tumor cell necrosis. Intracytoplasmic paranuclear hyaline inclusions may be occasionally seen. Inflammatory cells, such as lymphocytes and macrophages, are also frequently present [13].

By immunohistochemistry (IHC), DSRCTs express several markers of various lineages. An important diagnostic stain for DSRCTs is WT1 (Wilms Tumor 1), known to be positive in many of these tumors [13]. WT1 nuclear stain against the C terminus can be used to differentiate DSRCTs (WT1+) from Ewing sarcoma/primitive neuroectodermal tumors, neuroblastomas, or rhabdoid tumors of the kidney (WT1−) [26]. Additionally, DSRCTs are positive for several cytokeratins (CKs), such as CK7 and AE1/AE3, in addition to EMA (epithelial membrane antigen), which are typical of epithelial differentiation. Moreover, the expression of vimentin by a DSCRT displays its mesenchymal origin. The tumor also expresses desmin—a marker of myogenic differentiation—in a perinuclear dot-like pattern (cytoplasmic positivity due to perinuclear whorls of intermediate filaments [27]). In contrast to other small round cell tumors, DSRCTs do not typically express neuroendocrine markers such as synaptophysin and chromogranin [28] (Table 1). Nevertheless, Truong et al. demonstrated that androgen receptor (AR) and neuroendocrine genetic signatures appear to be inversely related in DSRCT cells [29,30].

Desmoplasia involves the formation of dense, fibrous tissue around tumor nests. It is a central characteristic of DSRCTs. The stroma functions as a structural element in addition to promoting local tumor development. It is characterized by various inflammatory cells such as lymphocytes and macrophages that together provide excellent conditions for tumor growth. These inflammatory cells can promote the survival and growth of the tumor evading their host’s immune system. Furthermore, the fibrous tissue serves as a scaffold to facilitate metastasis through local invasion and destruction of surrounding tissues and organs, contributing greatly to the aggressivity of this tumor [13].

Detecting desmoplasia in the diagnosis of DSCRTs is of great importance. The presence of this fibrotic response combined with its small cell morphology and specific IHC markers facilitates the distinction between a DSRCT and its mimickers. Treatment-wise, however, the presence of this dense stroma can make surgical removal of DSRCTs challenging. It can camouflage the margins around the tumor, resulting in failing to remove the tumor in its entirety [13]. Incomplete removal is associated with higher rates of metastasis and poorer prognosis in patients with DSRCTs [42]. Because desmoplasia is an important part of tumor progression, ongoing research attempts to target these stromal components. Therapeutic approaches may include targeted therapies or interventions to modulate the tumor microenvironment [43]. This may enhance both treatment efficacy and patient survival of DSRCTs [13,44,45].

Several studies and case reports contributed to our understanding of the clinical and histopathological features of DSRCTs of the GI tract. In one case report, a 6-year-old boy presented with a history of intermittent abdominal pain and episodic vomiting for the past 6 months that had become progressively more frequent. The right iliac fossa demonstrated a large heterogeneous enhanced mass outlined by imaging studies compressing adjacent bowel loops. Based on the core biopsy, the mass was originally diagnosed as a primitive neuroectodermal tumor. However, upon exploratory surgery, the mass was found to be arising from Meckel’s diverticulum. Histopathology demonstrated small round cells forming pseudo rosettes and a tumor locally invading its surrounding. IHC was positive for desmin and cytokeratin, supporting a diagnosis of DSRCT. The diagnosis was further supported by the presence of *EWS::WT1* gene fusion [14].

Takahira et al. reported a case of a previously healthy 27-year-old man with an intra-abdominal DSRCT that presented with a one-month history of abdominal pain, fullness, and constipation. The patient had a large peritoneal dissemination leading to colonic compression that ultimately resulted in bowel obstruction. Histological examination showed small, round cells with hyperchromatic nuclei located within a desmoplastic stroma. IHC was positive for the epithelial markers such as keratin and EMA, mesenchymal markers such as vimentin and desmin, as well as the neurofilaments marker S100 protein [46].

Therefore, characterizing the histopathology and IHC profile of a DSRCT in the GI tract and distinguishing it from its mimickers is crucial for accurate diagnosis.

## 5. Diagnosing DSRCTs in the GI Tract

Given the rare incidence and aggressive nature of DSRCTs, it is essential to accurately diagnose them and, therefore, treat them [47]. A study by Leça et al. shows the potential of fine-needle aspiration (FNA) and cytopathology in diagnosing DSRCTs. FNA smears show fragments of collagenous desmoplastic stroma and loosely cohesive small round cell clusters with positivity for WT1 and epithelial markers [48], making it a useful tool for rapid and cost-effective initial screening for DSRCTs. However, due to the challenging histomorphology of DSRCTs, IHC and molecular analysis may be required to confirm the diagnosis.

As mentioned above, DSRCT cells exhibit a polyphenotypic differentiation with co-expression of various epithelial, mesenchymal, and neural IHC markers [1], such as cytokeratins, vimentin, desmin, and WT1 [49]. This pattern of IHC reactivity is essential for differentiating between DSRCTs and other small round cell neoplasms, particularly when they develop outside of the usual sites such as the GI tract [22]. In a case of a DSRCT involving the stomach, IHC showed diffuse positivity for desmin and cytokeratin but also immunopositivity for WT1 [50]. Similarly, in another case of a DSRCT in the transverse colon, IHC revealed positive staining for cytokeratin, vimentin, desmin, and neural marker neuron-specific enolase (NSE) [47].

Lae et al. reported one of the largest case series including 32 DSRCTs, of which 81% were positive for desmin, 91% positive for WT1, 87% positive for keratin, 84% positive for NSE, 23% positive for CD99, and one case only was also positive for actin [51]. Another study by Barnoud et al. utilized a comparative approach, including DSRCTs and 71 other tumors, to assess whether WT1 by IHC is specific and sensitive for diagnosing DSRCTs and distinguishing them from other small round cell tumors. As a result, all the 15 DSRCTs (100%) demonstrated strong WT1 (C-19) nuclear immunoreactivity, while 71% of the Wilms tumors showed WT1 positivity nuclei, and only 2 out of 17 rhabdomyosarcomas demonstrated rare and focal nuclear positivity for WT1. Interestingly, none of the Ewing’s sarcoma/primitive neuroectodermal tumors (0 of 21), neuroblastomas (0 of 17), or rhabdoid tumors of the kidney (0 of 2) were positive for WT1 [26]. Given the available data, IHC allows for a more precise and definitive diagnosis when differentiating DSRCTs from their mimickers. The study by Arnold et al. focused on the challenges in differentiating DSRCT from Wilms tumor. Results showed that while desmin reactivity was more frequent in DSRCTs (11 of 12) than in Wilms tumor blastema (11 of 22), the associated dot-like and perinuclear cytoplasmic staining pattern is not specific to DSRCTs as it was seen in both DSRCTs and Wilms tumor blastema [33]. This finding elucidates the limitations of exclusively using IHC as a diagnostic tool for DSRCTs, emphasizing that detection of the characteristic *EWSR1::WT1* rearrangement along with the selective WT1 carboxy-terminus immunoreactivity remain the two complementary tools for accurately diagnosing DSRCTs [33].

The topic of WT1 carboxy-terminus immunoreactivity as a diagnostic marker is further expanded upon by Murphy et al., who examined one soft tissue DSRCT and five intra-abdominal DSRCTs. The soft tissue DSRCT was negative for WT1 C-terminal but positive with the N-terminal antibody [52]. The other five intra-abdominal DSRCT studies showed the expected nuclear staining with the WT1 C-terminal, but no reactivity for the N-terminal [52]. The findings in this study show that although most DSRCTs are positive for WT1 by IHC, some cases may have novel *EWS::WT1* fusion variant transcripts, resulting in atypical staining patterns [52].

As mentioned above, molecular studies play a crucial role in diagnosing DSCRTs and to differentiate them from their differential. In one study, *EWS::WT1* gene fusion transcript was detected in 29 of the 30 DSRCTs examined [51]. In another study by Wang et al., *EWSR1::WT1* gene fusion was present in all eight patients reported [13]. Therefore, the *EWSR1::WT1* fusion, which results from the t(11;22)(p13q12) translocation, serves as one of the most reliable tools for DSRCT diagnosis [1]. This applies to all DSRCTs of the GI tract, including a case of DSRCT of the Meckel’s diverticulum, where reverse transcriptase-polymerase chain reaction confirmed the presence of the *EWS::WT1* fusion gene [14].

This *EWSR1::WT1* fusion oncogene encodes for a chimeric protein that regulates transcription and thus serves as the driving source of the disease [1]. Mello et al. expanded on this by studying the downstream gene targets of this translocation. The study reports that this gene fusion upregulates the expression of PDGFRα, VEGF, and other proteins related to tumor and vascular cell proliferation [19]. Of note, the authors emphasized the importance of PDGFRα in the pathophysiology of the disease. PDGFRα plays a crucial role in the physiological healing process by inducing collagenous stromal production, inflammatory cell infiltration with macrophage chemotaxis predominance, neo-angiogenesis, and it works as a chemoattractant and inducer of proliferation for fibroblasts and endothelial cells [19]. Therefore, given that the *EWS::WT1* transcription factor translocation induces the unregulated expression of PDGFRα, this gene product may explain the characteristic histological findings of profuse desmoplastic stromal reaction and the increased vascularity seen in DSCRTs [19].

The homogenous presence of the *EWSR1::WT1* fusion oncogene in DSRCTs can be detected by several molecular studies, such as Next Generation Sequencing (NGS), which is the most reliable diagnostic tool available [53], or RT-PCR using paraffin-embedded tissue sections [49]. Given its widespread use in the clinical setting, the latter tool allows for a fast and cost-effective modality for diagnosing DSRCTs compared to NGS. While the DSRCT is unique for harboring the *EWSR1::WT1* gene fusion, its differential diagnosis also has specific genetic signatures: *PAX3::FOXO1*, *PAX7::FOXO1*, or *PAX3::AFX* gene fusions for alveolar rhabdomyosarcoma, *EWSR1::FLI1*, *EWSR1::ERG*, *EWSR1::FEV*, *EWSR1::ETV1*, *EWSR1::E1AF*, *EWSR1::ZSG*, or *FUS::ERG* gene fusions for Ewing sarcoma/peripheral neuroectodermal tumor, and *CIC::DUX44* or *BCOR::CCNB35* gene fusions for undifferentiated round cell sarcomas [54].

Imaging modalities such as CT, MRI, and/or PET play a critical role in managing DSRCTs. The standard imaging modalities used for staging and assessing for remission/relapse in DSRCTs include CT scans and MRI. By CT and MRI, DSRCTs typically show heterogeneous soft-tissue enhancement with cystic degeneration [55]. In a study of 65 DSRCTs, the most common CT finding was multiple peritoneal soft tissue masses and a larger dominant mass located in the rectovesical or rectouterine space in more than half of the cases [56]. It was also reported that 40% of the patients had metastatic disease to the liver, lungs, spleen, or bones at the time of diagnosis, reflecting the aggressiveness of the disease [56]. In other studies, up to 80% of DSRCT patients had metastatic disease at diagnosis, with the two most common metastatic sites being the liver (33%) and the lungs (21%) [57].

Given that DSRCTs are metabolically active, Ostermeier et al. evaluated the use of FDG PET/CT imaging on eight patients and identified increased metabolic activity in all patients studied, demonstrating its utility in initial staging, monitoring treatment response, and even surveillance for recurrence [58]. This was further validated in a larger study by Arora et al. including 65 patients with DSRCTs, of which 11 received FDG PET/CT scans and showed that FDG PET/CT accurately detected 97.4% of all DSRCT lesions [56].

All in all, a multimodal approach incorporating clinical history and physical examination, histopathology, IHC, molecular diagnostics, and appropriate imaging are all needed to diagnose and manage DSRCTs of the GI tract. These diagnostic techniques collectively and combined help in accurately diagnosing DSRCTs and ruling out differential mimics.

## 6. Prognostic Factors of DSRCTs of the GI Tract

As is the case with most cancers, the key prognostic factors for DSRCTs include tumor size, location, and the presence of metastasis [57]. In general, patients with extra-abdominal DSRCTs live longer than those with DSRCTs of the GI tract [57]. Patients with non-metastatic intra-abdominal DSRCTs who undergo surgical resection with negative margins have a median survival of 47 months compared to 16 months for patients who do not receive surgery [57]. Further proof that surgical intervention serves as a favorable prognostic factor by increasing survival time after diagnosis was highlighted in a study on DSRCT patients of the GI tracts. Those who underwent surgical resection had a median survival of 34 months, while those who did not had a mean survival of 14 months [59]. Additionally, patients who received consolidative cytoreductive surgery with hyperthermic intraperitoneal chemotherapy had a longer survival time (30.6 versus 11.2 months) [4]. Radiation therapy also plays a role in locoregional control in patients with metastatic intra-abdominal tumors, or those with positive surgical margins. Basically, radiation therapy can help reduce the risk of local recurrence by targeting the microscopic disease that surgery could not eliminate. A positive correlation was found between radiation therapy treatment and survival time, with a median survival of 47 months for patients who received radiation versus 14 months for those who did not [57]. The three-year survival rate was shown to increase from 37.6% to 61.2% with postoperative radiation and chemotherapy [60].

There was no significant difference, however, in median survival when evaluating age, gender, or tumor size [57]. Nonetheless, DSRCTs are aggressive neoplasms with poor prognosis, particularly when they arise in the GI tract. A study revealed that 19 out of 27 DSRCT patients died due to uncontrolled local or widespread metastasis 3 to 46 months after diagnosis, with a mean survival of 20 months only [51], reinforcing that although current therapies can only slightly extend survival time, remission and disease-free survival rates remain low. DSRCTs of the GI tract have a high likelihood of diffuse peritoneal dissemination. Unfortunately, DSRCTs in the stomach and transverse colon can be large, locally invasive, and often difficult to resect completely [61]. There are not many studies on the significance of lymph node involvement in DSRCTs [62,63]. One reported case of a young female with a DSRCT arising in the stomach demonstrated the tumor’s aggressive nature in association with lymph node positivity and recurrence [47].

## 7. Treatment Approaches and Clinical Outcomes of DSRCTs of the GI Tract

Survival rates for DSRCTs vary based on several factors such as disease stage, response to treatment, and recurrence of disease. Time of diagnosis and presence of metastasis at diagnosis significantly affects the prognosis. Early detection of DSRCTs of the GI tract is usually difficult as patients present with regular GI symptoms that do not raise suspicion for a malignancy, and it is not until the tumor grows enough in size that it causes prominent clinical signs and persisting symptoms prompting a serious medical workup and intervention [64]. In such cases, patients usually endure signs of ileus, urinary tract compression with a distended bladder, enuresis, and other mechanical mass compression signs [46].

Treatment approaches for DSRCTs, particularly those of the GI tract, often involve a combination of surgery, chemotherapy, and radiation therapy. Chemotherapy is frequently used initially and alongside surgery to target any remaining microscopic cancer cells or treat tumors that cannot be surgically removed entirely. Surgery aims to remove as much of the tumor as possible, but its effectiveness depends on the tumor’s location and size. Radiation therapy may also be employed to target residual disease or manage symptoms when surgery is not feasible; however, the toxicities that follow have limited its benefits. Therefore, a multidisciplinary approach that integrates surgery, chemotherapy, and radiation therapy is typically required to achieve optimal results. When these modalities are used in a multimodal therapy regimen, it was found to have a three-year survival rate of up to 55% [65].

### 7.1. Chemotherapy

Many aggressive treatment modalities have been used in treating DSRCTs; however, the remission rate remains low. The nature of a DSRCT being highly metastatic makes it a great candidate for initial treatment with chemotherapy. Prior to chemotherapy, however, the marker NSE may be used to determine the progress the patient makes post-chemotherapy session [46].

Usually, the regimen used contains ifosfamide and doxorubicin, both of which are used for soft tissue sarcomas, whether it is the “P6 regimen” consisting of cyclophosphamide, doxorubicin, vincristine, ifosfamide, and etoposide, or the “VAIA regimen” consisting of vincristine, dactinomycin, ifosfamide, and doxorubicin. The “P6 regimen” was established for treating Ewing sarcoma [66]. A common second-line regimen combines cyclophosphamide and topotecan. A chemotherapy regimen found to be more effective in children uses cyclophosphamide or ifosfamide in addition to vincristine and doxorubicin in conjunction with full abdominal resection and radiation of the masses for the best post-treatment chances [67]. Chemotherapy also helps decrease the vascularity of tumors and improves malignant ascites.

### 7.2. Surgical

Surgical resection of resectable masses significantly reduces relapse rates. Patients typically receive chemotherapy prior to surgery until their response plateaus. Hayes-Jordan et al. recommend waiting at least 4 months during which systemic chemotherapy is given before evaluating for surgical candidacy [68]. Whether a patient qualifies for surgery depends on various factors; for example, those with extra-abdominal metastases often forego resection due to high recurrence and mortality risks [69]. This issue has sparked controversy regarding the appropriateness of resection, exacerbated by variability between cases and uncertainty regarding pre- versus post-chemotherapy extra-abdominal relapse [66].

When patients proceed to resection, prognosis significantly improves. Lal et al. demonstrated a 58% 3-year survival rate among patients undergoing complete tumor resection post-chemotherapy, compared to 0% for those who did not undergo resection [65,70].

### 7.3. Radiation

Radiation therapy has been used in multidisciplinary regimens as well as independently in DSRCTs. Whole abdominopelvic radiation therapy (WAP-RT) has been studied for its effectiveness in reducing local recurrence; however, its utility is constrained by associated toxicities such as leukopenia, thrombocytopenia, anemia, and small bowel obstruction.

A retrospective study spanning from 1992 to 2001 investigated the use of whole abdominopelvic irradiation (WAPI) following chemotherapy and surgical resection, revealing a 3-year survival rate of 48%. Notably, all patients in this study experienced hematological toxicities and small bowel obstruction [71]. Intensity-modulated radiation therapy (IMRT) has been explored for its potential benefits as well. A retrospective study involving 31 patients from 1992 to 2011 compared IMRT to 2-dimensional radiation therapy (2D-RT), demonstrating reduced toxicity [72], especially hematological toxicities.

Combining IMRT with WAP as a unified treatment approach has been further studied due to reduced toxicity observed in several cases. A retrospective analysis encompassing all patients treated with WAP-IMRT from 2006 to 2010 found that this approach, when combined with radio-sensitizing chemotherapy, was better tolerated compared to WAP-RT in terms of toxicities [73].

## 8. Clinical Trials and Future Perspectives

Emerging therapies and clinical trials offer hope for more effective treatments with fewer side effects. Advances in targeted therapies, immunotherapy, and novel chemotherapy agents are under exploration. Participation in clinical trials provides access to cutting-edge treatments and contributes to the advancement of care for DSRCTs. Additionally, a multidisciplinary approach remains vital in managing DSRCTs, ensuring a comprehensive treatment strategy to address all aspects of the disease and optimize patient outcomes.

Hyperthermic intraperitoneal perfusion (HIPEC) is a new emerging treatment modality that was first reported in 2004 and subsequently in 2007, primarily for its efficacy in eliminating microscopic disease post-complete resection [66]. In one study, HIPEC was used adjunctively after complete tumor resection in 8 out of 24 patients, achieving a notable 71% 3-year survival rate compared to 26% in other experimental groups [74]. However, HIPEC use also led to morbidity, including renal insufficiency and gastroparesis in some patients [66,74].

Research into immune and biological targeted therapies is ongoing. Theoretically, potential targeted therapies include addressing VEGF inhibition, androgen receptor activity, and agents affecting the connective tissue growth factor CCN2 and GD2 glycosphingolipid receptors [75,76].

Radioimmunotherapy (RIT) using the murine monoclonal antibody 131I-omburtamab targeting antigen B7-H3 has progressed to phase 1 trials, demonstrating low radiation exposure in intraperitoneal administration and warranting further investigation in combination with multimodal therapies [77]. B7-H3 (CD276) is a member of the B7 family of immunoregulatory proteins. It is a type I transmembrane glycoprotein involved in immune response modulation, particularly in tumor immunity. B7-H3 is commonly overexpressed in many solid tumors, including DSRCTs [78], making it a potential target for therapies. In DSRCTs, B7-H3 contributes to immune evasion by inhibiting the activation and proliferation of T cells, facilitating tumor survival in an immunosuppressive microenvironment. While B7-H3 is not exclusive to DSRCTs, its high expression in these tumors suggests potential utility as a biomarker, as well as a target for monoclonal antibodies or antibody-drug conjugates (e.g., enoblituzumab, 8H9) [79,80].

Clinically, anticancer agents such as trabectedin [81] have been explored in clinical trials and off-label used for relapsed DSRCT patients [82]. Trabectedin, along with its derivative Lurbinectedin which is also under investigation in clinical trials, is thought to inhibit the *EWS::WT1* transcription factor [70].

Given the high risk of relapse, most patients undergo systemic treatment across multiple periods, potentially impacting their quality of life during and after treatment.

## 9. Conclusions

In conclusion, DSRCTs of the GI tract represent a unique and challenging variant of soft tissue sarcomas. In this review, we elaborate on the biological behavior of those tumors, their origin and pathophysiology, molecular signatures, and the critical role of comprehensive diagnostic approaches that integrate histopathological evaluation with advanced IHC and molecular techniques. After accurately diagnosing DSRCTs, a collaborative effort among pathologists, oncologists, and surgical teams is needed to optimize management strategies and improve patient outcomes.

## Figures and Tables

**Table 1:** Biomarkers and genetic signatures for distinguishing DSRCTs, Ewing sarcoma, and alveolar rhabdomyosarcoma.

IHC Markers and Genetic Signatures	DSRCT	Ewing Sarcoma	Alveolar Rhabdomyosarcoma
EMA and keratins [31]	Variable (distinctive dot-like cytoplasmic expression occasionally)	Negative (but diffuse in adamantinoma-like variant [32])	Variable (may show focal keratin positivity)
Desmin	Distinct dot-like and perinuclear cytoplasmic staining pattern) [27]	Negative	Positive
Vimentin	Distinctive dot-like cytoplasmic expression occasionally [11]	Positive in 80–90%	Negative
WT1 (C terminus)	Positive in 65–90% [33] but negative for WT1 (N terminus)	Negative	Negative
NSE, neurofilament, and synaptophysin (NE markers)	Variable [34,35]	Variable	Variable
Actin	Variable	Negative	Positive
Myogenin [36,37]	Negative	Negative	Positive
MyoD1 [36]	Negative	Negative	Positive
CD99	Negative	Positive (membranous; up to 95%) [38]	Positive
FLI1	Negative	Positive (nuclear with *EWSR1::FLI1* fusion; up to 90%) [39]	Negative
ERG	Negative	Positive (nuclear with *EWSR1::ERG* fusion) [40]	Negative
NKX2.2	Negative	Positive (nuclear; up to 93%) [41]	Negative
S100 protein [11]	Negative	Negative	Negative
Genetic signatures	*EWSR1::WT1* gene fusion	*EWSR1* rearrangements: *EWSR1::FLI1*, *EWSR1::ERG*, *EWSR1::FEV*, *EWSR1::ETV1*, *EWSR1::E1AF*, *EWSR1::ZSG*, or *FUS::ERG* gene fusions	t(2;13) or t(1;13) involving *FOXO1* and *PAX3* or *PAX7*: *PAX3::FOXO1*, *PAX7::FOXO1*, or *PAX3::AFX* gene fusions

Abbreviations: *AFX*: forkhead transcription factor gene; DSRCT: desmoplastic small round cell tumor; *E1AF*: adenovirus E1A enhancer-binding gene; EMA: epithelial membrane antigen; *ERG*: ETS-related gene; *ETV1*: ETS variant transcription factor 1 gene; *EWSR1*: Ewing sarcoma breakpoint region 1; *FEV*: human fifth Ewing variant gene; *FLI1*: friend leukemia integration 1 gene; *FOXO1*: forkhead box protein O1 gene; *FUS*: fused in sarcoma gene; IHC: immunohistochemistry; MyoD1: myogenic differentiation 1; NE: neuroendocrine; NSE: neuron-specific enolase; *PAX3*: paired box 3 gene; *PAX7*: paired box 7 gene; WT1: Wilms tumor protein 1; *ZSG*: zinc finger sarcoma gene.

## References

[1] Hendricks A., Boerner K., Germer C.T., Wiegering A. (2021). Desmoplastic Small Round Cell Tumors: A review with focus on clinical management and therapeutic options. Cancer Treat. Rev..

[2] Gerald W.L., Rosai J. (1989). Case 2. Desmoplastic small cell tumor with divergent differentiation. Pediatr. Pathol..

[3] Ordóñez N.G. (1998). Desmoplastic small round cell tumor: I: A histopathologic study of 39 cases with emphasis on unusual histological patterns. Am. J. Surg. Pathol..

[4] Jayakrishnan T., Moll R., Sandhu A., Sanguino A., Kaur G., Mao S. (2021). Desmoplastic Small Round-cell Tumor: Retrospective Review of Institutional Data and Literature Review. Anticancer Res..

[5] Jordan A.H., Pappo A. (2012). Management of desmoplastic small round-cell tumors in children and young adults. J. Pediatr. Hematol. Oncol..

[6] Lin P.P., Jaffe N., Herzog C.E., Costelloe C.M., Deavers M.T., Kelly J.S., Patel S.R., Madewell J.E., Lewis V.O., Cannon C.P. (2007). Chemotherapy response is an important predictor of local recurrence in Ewing sarcoma. Cancer.

[7] Smith M.A., Altekruse S.F., Adamson P.C., Reaman G.H., Seibel N.L. (2014). Declining childhood and adolescent cancer mortality. Cancer.

[8] PDQ Pediatric Treatment Editorial Board (2002). Ewing Sarcoma and Undifferentiated Small Round Cell Sarcomas of Bone and Soft Tissue Treatment (PDQ®): Health Professional Version. PDQ Cancer Information Summaries.

[9] Bourcier K., Le Cesne A., Tselikas L., Adam J., Mir O., Honore C., de Baere T. (2019). Basic Knowledge in Soft Tissue Sarcoma. Cardiovasc. Interv. Radiol..

[10] Cidre-Aranaz F., Watson S., Amatruda J.F., Nakamura T., Delattre O., de Alava E., Dirksen U., Grünewald T.G.P. (2022). Small round cell sarcomas. Nat. Rev. Dis. Primers.

[11] Cummings O.W., Ulbright T.M., Young R.H., Dei Tos A.P., Fletcher C.D., Hull M.T. (1997). Desmoplastic small round cell tumors of the paratesticular region. A report of six cases. Am. J. Surg. Pathol..

[12] Fois A.G., Pirina P., Arcadu A., Becciu F., Manca S., Marras V., Canu S., Castagna G., Ginesu G.C., Zinellu A. (2017). Desmoplastic small round cell tumors of the pleura: A review of the clinical literature. Multidiscip. Respir. Med..

[13] Wang L.L., Ji Z.H., Gao Y., Chang H., Sun P.P., Li Y. (2021). Clinicopathological features of desmoplastic small round cell tumors: Clinical series and literature review. World J. Surg. Oncol..

[14] Qureshi S.S., Ramadwar M.R., Viswanathan S., Bakshi A.V., Arora B., Gupta T., Laskar S., Medhi S.S., Muckaden M.A., Banavali S.D. (2007). Desmoplastic small round cell tumor of Meckels diverticulum. J. Clin. Oncol..

[15] Magrath J.W., Sampath S.S., Flinchum D.A., Hartono A.B., Goldberg I.N., Boehling J.R., Savkovic S.D., Lee S.B. (2024). Comprehensive Transcriptomic Analysis of EWSR1::WT1 Targets Identifies CDK4/6 Inhibitors as an Effective Therapy for Desmoplastic Small Round Cell Tumors. Cancer Res..

[16] Schoolmeester J.K., Folpe A.L., Nair A.A., Halling K., Sutton B.C., Landers E., Karnezis A.N., Dickson B.C., Nucci M.R., Kolin D.L. (2021). EWSR1-WT1 gene fusions in neoplasms other than desmoplastic small round cell tumor: A report of three unusual tumors involving the female genital tract and review of the literature. Mod. Pathol..

[17] Bandopadhayay P., Jabbour A.M., Riffkin C., Salmanidis M., Gordon L., Popovski D., Rigby L., Ashley D.M., Watkins D.N., Thomas D.M. (2013). The oncogenic properties of EWS/WT1 of desmoplastic small round cell tumors are unmasked by loss of p53 in murine embryonic fibroblasts. BMC Cancer.

[18] de Matos B.M., Robert A.W., Stimamiglio M.A., Correa A. (2022). Pluripotent-derived Mesenchymal Stem/stromal Cells: An Overview of the Derivation Protocol Efficacies and the Differences Among the Derived Cells. Stem Cell Rev. Rep..

[19] Mello C.A., Campos F.A.B., Santos T.G., Silva M.L.G., Torrezan G.T., Costa F.D., Formiga M.N., Nicolau U., Nascimento A.G., Silva C. (2021). Desmoplastic Small Round Cell Tumor: A Review of Main Molecular Abnormalities and Emerging Therapy. Cancers.

[20] D’Souza N., Rossignoli F., Golinelli G., Grisendi G., Spano C., Candini O., Osturu S., Catani F., Paolucci P., Horwitz E.M. (2015). Mesenchymal stem/stromal cells as a delivery platform in cell and gene therapies. BMC Med..

[21] Grünewald T.G.P., Cidre-Aranaz F., Surdez D., Tomazou E.M., de Álava E., Kovar H., Sorensen P.H., Delattre O., Dirksen U. (2018). Ewing sarcoma. Nat. Rev. Dis. Primers.

[22] Dagher R., Helman L. (1999). Rhabdomyosarcoma: An overview. Oncologist.

[23] Ponzoni M., Bachetti T., Corrias M.V., Brignole C., Pastorino F., Calarco E., Bensa V., Giusto E., Ceccherini I., Perri P. (2022). Recent advances in the developmental origin of neuroblastoma: An overview. J. Exp. Clin. Cancer Res..

[24] Shawraba F., Hammoud H., Mrad Y., Saker Z., Fares Y., Harati H., Bahmad H.F., Nabha S. (2021). Biomarkers in Neuroblastoma: An Insight into Their Potential Diagnostic and Prognostic Utilities. Curr. Treat. Options Oncol..

[25] Pangua C., Rogado J., Serrano-Montero G., Belda-Sanchís J., Álvarez Rodríguez B., Torrado L., Rodríguez De Dios N., Mielgo-Rubio X., Trujillo J.C., Couñago F. (2022). New perspectives in the management of small cell lung cancer. World J. Clin. Oncol..

[26] Barnoud R., Sabourin J.C., Pasquier D., Ranchère D., Bailly C., Terrier-Lacombe M.J., Pasquier B. (2000). Immunohistochemical expression of WT1 by desmoplastic small round cell tumor: A comparative study with other small round cell tumors. Am. J. Surg. Pathol..

[27] Chang F. (2006). Desmoplastic small round cell tumors: Cytologic, histologic, and immunohistochemical features. Arch. Pathol. Lab. Med..

[28] Slotkin E.K., Bowman A.S., Levine M.F., Dela Cruz F., Coutinho D.F., Sanchez G.I., Rosales N., Modak S., Tap W.D., Gounder M.M. (2021). Comprehensive Molecular Profiling of Desmoplastic Small Round Cell Tumor. Mol. Cancer Res..

[29] Truong D., Lamhamedi-Cherradi S.-E., Maitituoheti M., Beird H.C., Arslan E., Wu C.-C., Krishnan S., Ingram D., Futreal P.A., Titus M. (2023). The epigenetic impact and therapeutic opportunity of AR-directed therapy for DSRCT. Cancer Res..

[30] Tam Y.B., Jones R.L., Huang P.H. (2023). Molecular profiling in desmoplastic small round cell tumours. Int. J. Biochem. Cell Biol..

[31] Gerald W.L., Miller H.K., Battifora H., Miettinen M., Silva E.G., Rosai J. (1991). Intra-abdominal desmoplastic small round-cell tumor. Report of 19 cases of a distinctive type of high-grade polyphenotypic malignancy affecting young individuals. Am. J. Surg. Pathol..

[32] Yoshida A. (2023). Ewing and Ewing-like sarcomas: A morphological guide through genetically-defined entities. Pathol. Int..

[33] Arnold M.A., Schoenfield L., Limketkai B.N., Arnold C.A. (2014). Diagnostic pitfalls of differentiating desmoplastic small round cell tumor (DSRCT) from Wilms tumor (WT): Overlapping morphologic and immunohistochemical features. Am. J. Surg. Pathol..

[34] Yachnis A.T., Rorke L.B., Biegel J.A., Perilongo G., Zimmerman R.A., Sutton L.N. (1992). Desmoplastic primitive neuroectodermal tumor with divergent differentiation. Broadening the spectrum of desmoplastic infantile neuroepithelial tumors. Am. J. Surg. Pathol..

[35] Trikalinos N.A., Chrisinger J.S.A., Van Tine B.A. (2021). Common Pitfalls in Ewing Sarcoma and Desmoplastic Small Round Cell Tumor Diagnosis Seen in a Study of 115 Cases. Med. Sci..

[36] Gerald W.L., Ladanyi M., de Alava E., Cuatrecasas M., Kushner B.H., LaQuaglia M.P., Rosai J. (1998). Clinical, pathologic, and molecular spectrum of tumors associated with t(11;22)(p13;q12): Desmoplastic small round-cell tumor and its variants. J. Clin. Oncol..

[37] Rekhi B., Gupta C., Chinnaswamy G., Qureshi S., Vora T., Khanna N., Laskar S. (2018). Clinicopathologic features of 300 rhabdomyosarcomas with emphasis upon differential expression of skeletal muscle specific markers in the various subtypes: A single institutional experience. Ann. Diagn. Pathol..

[38] Folpe A.L., Goldblum J.R., Rubin B.P., Shehata B.M., Liu W., Dei Tos A.P., Weiss S.W. (2005). Morphologic and immunophenotypic diversity in Ewing family tumors: A study of 66 genetically confirmed cases. Am. J. Surg. Pathol..

[39] Folpe A.L., Hill C.E., Parham D.M., O’Shea P.A., Weiss S.W. (2000). Immunohistochemical detection of FLI-1 protein expression: A study of 132 round cell tumors with emphasis on CD99-positive mimics of Ewing’s sarcoma/primitive neuroectodermal tumor. Am. J. Surg. Pathol..

[40] Wang W.L., Patel N.R., Caragea M., Hogendoorn P.C., López-Terrada D., Hornick J.L., Lazar A.J. (2012). Expression of ERG, an Ets family transcription factor, identifies ERG-rearranged Ewing sarcoma. Mod. Pathol..

[41] Yoshida A., Sekine S., Tsuta K., Fukayama M., Furuta K., Tsuda H. (2012). NKX2.2 is a useful immunohistochemical marker for Ewing sarcoma. Am. J. Surg. Pathol..

[42] Xiang T., Zhang S.Y., Wang S.S., Fei R.S., Li H. (2020). A nationwide analysis of desmoplastic small round cell tumor. Medicine.

[43] Zhang T., Febres-Aldana C.A., Liu Z., Dix J.M., Cheng R., Dematteo R.G., Lui A.J.W., Khodos I., Gili L., Mattar M.S. (2024). HER2 antibody-drug conjugates are active against desmoplastic small round cell tumor. Clin. Cancer Res..

[44] Magrath J.W., Goldberg I.N., Truong D.D., Hartono A.B., Sampath S.S., Jackson C.E., Ghosh A., Cardin D.L., Zhang H., Ludwig J.A. (2024). Enzalutamide induces cytotoxicity in desmoplastic small round cell tumor independent of the androgen receptor. Commun. Biol..

[45] Zhu L., Keck J., Stommel J., Johnson B., Corless C.L., Ryan C.W., Mills G.B., Davis L.E. (2023). Deep multi-omic analyses to identify targetable pathways in desmoplastic small round cell tumor (DSRCT) with opportunities for clinical intervention. J. Clin. Oncol..

[46] Takahira K., Ohi S., Fujii N., Matsuura Y., Sano M., Hanai H., Kaneko E. (2000). Intra-abdominal desmoplastic small round cell tumor (IDSRT). J. Gastroenterol..

[47] Abu-Zaid A., Azzam A., Alnajjar A., Al-Hussaini H., Amin T. (2013). Desmoplastic small round cell tumor of stomach. Case Rep. Gastrointest. Med..

[48] Leça L.B., Vieira J., Teixeira M.R., Monteiro P. (2012). Desmoplastic small round cell tumor: Diagnosis by fine-needle aspiration cytology. Acta Cytol..

[49] Lee Y.S., Hsiao C.H. (2007). Desmoplastic small round cell tumor: A clinicopathologic, immunohistochemical and molecular study of four patients. J. Formos. Med. Assoc..

[50] Huang J., Sha L., Zhang H., Tang X., Zhang X. (2015). Desmoplastic small round cell tumor in transverse colon: Report of a rare case. Int. Surg..

[51] Lae M.E., Roche P.C., Jin L., Lloyd R.V., Nascimento A.G. (2002). Desmoplastic small round cell tumor: A clinicopathologic, immunohistochemical, and molecular study of 32 tumors. Am. J. Surg. Pathol..

[52] Murphy A.J., Bishop K., Pereira C., Chilton-MacNeill S., Ho M., Zielenska M., Thorner P.S. (2008). A new molecular variant of desmoplastic small round cell tumor: Significance of WT1 immunostaining in this entity. Hum. Pathol..

[53] Desai A.N., Kurian C.J., Rafferty W., Behrens D.L., Khrizman P. (2024). Case report: An unusual presentation of intra-abdominal desmoplastic small round cell tumor. Front. Oncol..

[54] Dunlop H.M., Bende B., Ruff S.M., Kim A., Fisher J.L., Grignol V.P., Contreras C.M., Obeng-Gyasi S., Konieczkowski D.J., Pawlik T.M. (2024). Disparities in Survival and NCCN Guideline–Concordant Care in Patients With Extremity Soft Tissue Sarcoma. J. Natl. Compr. Cancer Netw..

[55] Thomas R., Rajeswaran G., Thway K., Benson C., Shahabuddin K., Moskovic E. (2013). Desmoplastic small round cell tumour: The radiological, pathological and clinical features. Insights Imaging.

[56] Arora V.C., Price A.P., Fleming S., Sohn M.J., Magnan H., LaQuaglia M.P., Abramson S. (2013). Characteristic imaging features of desmoplastic small round cell tumour. Pediatr. Radiol..

[57] Wong H.H., Hatcher H.M., Benson C., Al-Muderis O., Horan G., Fisher C., Earl H.M., Judson I. (2013). Desmoplastic small round cell tumour: Characteristics and prognostic factors of 41 patients and review of the literature. Clin. Sarcoma Res..

[58] Ostermeier A., McCarville M.B., Navid F., Snyder S.E., Shulkin B.L. (2015). FDG PET/CT imaging of desmoplastic small round cell tumor: Findings at staging, during treatment and at follow-up. Pediatr. Radiol..

[59] Hassan I., Shyyan R., Donohue J.H., Edmonson J.H., Gunderson L.L., Moir C.R., Arndt C.A., Nascimento A.G., Que F.G. (2005). Intraabdominal desmoplastic small round cell tumors: A diagnostic and therapeutic challenge. Cancer.

[60] Atallah V., Honore C., Orbach D., Helfre S., Ducassou A., Thomas L., Levitchi M.B., Mervoyer A., Naji S., Dupin C. (2016). Role of Adjuvant Radiation Therapy After Surgery for Abdominal Desmoplastic Small Round Cell Tumors. Int. J. Radiat. Oncol. Biol. Phys..

[61] Lai B., Siyi L., Zhou J., Cui L. (2024). Desmoplastic small round-cell tumor. Asian J. Surg..

[62] Backer A., Mount S.L., Zarka M.A., Trask C.E., Allen E.F., Gerald W.L., Sanders D.A., Weaver D.L. (1998). Desmoplastic small round cell tumour of unknown primary origin with lymph node and lung metastases: Histological, cytological, ultrastructural, cytogenetic and molecular findings. Virchows Arch..

[63] Faras F., Abo-Alhassan F., Hussain A.H., Sebire N.J., Al-Terki A.E. (2015). Primary desmoplastic small round cell tumor of upper cervical lymph nodes. Oral Surg. Oral Med. Oral Pathol. Oral Radiol..

[64] Tsoukalas N., Kiakou M., Nakos G., Tolia M., Galanopoulos M., Tsapakidis K., Kamposioras K., Christofyllakis C., Dimitrakopoulos G., Sambaziotis D. (2020). Desmoplastic small round-cell tumour of the peritoneal cavity: Case report and literature review. Ann. R. Coll. Surg. Engl..

[65] Lal D.R., Su W.T., Wolden S.L., Loh K.C., Modak S., La Quaglia M.P. (2005). Results of multimodal treatment for desmoplastic small round cell tumors. J. Pediatr. Surg..

[66] Reijers S.J.M., Siew C.C.H., Kok N.F.M., Honoré C., van Houdt W.J. (2023). Intra-Abdominal Desmoplastic Small Round Cell Tumor (DSRCT) and the Role of Hyperthermic Intraperitoneal Chemotherapy (HIPEC): A Review. Curr. Oncol..

[67] Hayes-Jordan A., LaQuaglia M.P., Modak S. (2016). Management of desmoplastic small round cell tumor. Semin. Pediatr. Surg..

[68] Hayes-Jordan A.A., Coakley B.A., Green H.L., Xiao L., Fournier K.F., Herzog C.E., Ludwig J.A., McAleer M.F., Anderson P.M., Huh W.W. (2018). Desmoplastic Small Round Cell Tumor Treated with Cytoreductive Surgery and Hyperthermic Intraperitoneal Chemotherapy: Results of a Phase 2 Trial. Ann. Surg. Oncol..

[69] Hayes-Jordan A. (2015). Cytoreductive Surgery Followed by Hyperthermic Intraperitoneal Chemotherapy in DSRCT: Progress and Pitfalls. Curr. Oncol. Rep..

[70] Hovsepyan S., Giani C., Pasquali S., Di Giannatale A., Chiaravalli S., Colombo C., Orbach D., Bergamaschi L., Vennarini S., Gatz S.A. (2023). Desmoplastic small round cell tumor: From state of the art to future clinical prospects. Expert Rev. Anticancer Ther..

[71] Goodman K.A., Wolden S.L., La Quaglia M.P., Kushner B.H. (2002). Whole abdominopelvic radiotherapy for desmoplastic small round-cell tumor. Int. J. Radiat. Oncol. Biol. Phys..

[72] Desai N.B., Stein N.F., LaQuaglia M.P., Alektiar K.M., Kushner B.H., Modak S., Magnan H.M., Goodman K., Wolden S.L. (2013). Reduced toxicity with intensity modulated radiation therapy (IMRT) for desmoplastic small round cell tumor (DSRCT): An update on the whole abdominopelvic radiation therapy (WAP-RT) experience. Int. J. Radiat. Oncol. Biol. Phys..

[73] Pinnix C.C., Fontanilla H.P., Hayes-Jordan A., Subbiah V., Bilton S.D., Chang E.L., Grosshans D.R., McAleer M.F., Sulman E.P., Woo S.Y. (2012). Whole abdominopelvic intensity-modulated radiation therapy for desmoplastic small round cell tumor after surgery. Int. J. Radiat. Oncol. Biol. Phys..

[74] Hayes-Jordan A., Green H., Fitzgerald N., Xiao L., Anderson P. (2010). Novel treatment for desmoplastic small round cell tumor: Hyperthermic intraperitoneal perfusion. J. Pediatr. Surg..

[75] Loktev A., Shipley J.M. (2020). Desmoplastic small round cell tumor (DSRCT): Emerging therapeutic targets and future directions for potential therapies. Expert Opin. Ther. Targets.

[76] Yamamoto Y., Loriot Y., Beraldi E., Zhang F., Wyatt A.W., Al Nakouzi N., Mo F., Zhou T., Kim Y., Monia B.P. (2015). Generation 2.5 antisense oligonucleotides targeting the androgen receptor and its splice variants suppress enzalutamide-resistant prostate cancer cell growth. Clin. Cancer Res..

[77] Modak S., Zanzonico P., Grkovski M., Slotkin E.K., Carrasquillo J.A., Lyashchenko S.K., Lewis J.S., Cheung I.Y., Heaton T., LaQuaglia M.P. (2020). B7H3-Directed Intraperitoneal Radioimmunotherapy With Radioiodinated Omburtamab for Desmoplastic Small Round Cell Tumor and Other Peritoneal Tumors: Results of a Phase I Study. J. Clin. Oncol..

[78] Hingorani P., Dinu V., Zhang X., Lei H., Shern J.F., Park J., Steel J., Rauf F., Parham D., Gastier-Foster J. (2020). Transcriptome analysis of desmoplastic small round cell tumors identifies actionable therapeutic targets: A report from the Children’s Oncology Group. Sci. Rep..

[79] Espinosa-Cotton M., Guo H.F., Tickoo S.K., Cheung N.V. (2023). Identification of immunotherapy and radioimmunotherapy targets on desmoplastic small round cell tumors. Front. Oncol..

[80] Magrath J.W., Espinosa-Cotton M., Flinchum D.A., Sampath S.S., Cheung N.K., Lee S.B. (2024). Desmoplastic small round cell tumor: From genomics to targets, potential paths to future therapeutics. Front. Cell Dev. Biol..

[81] Uboldi S., Craparotta I., Colella G., Ronchetti E., Beltrame L., Vicario S., Marchini S., Panini N., Dagrada G., Bozzi F. (2017). Mechanism of action of trabectedin in desmoplastic small round cell tumor cells. BMC Cancer.

[82] Emambux S., Kind M., Le Loarer F., Toulmonde M., Stoeckle E., Italiano A. (2017). Clinical activity of eribulin in advanced desmoplastic small round-cell tumor. Anticancer Drugs.

